# Morphometric study of the bony labyrinth of the inner ear in the European moles *Talpa europaea*, *Talpa occidentalis*, and *Talpa aquitania*

**DOI:** 10.1111/joa.70017

**Published:** 2025-07-03

**Authors:** Alice Melekian, Sergio Daniel Tarquini, Violaine Nicolas, Nathalie Poulet‐Crovisier, Sandrine Ladevèze

**Affiliations:** ^1^ CR2P–Centre de Recherche en Paléontologie Paris UMR 7207, Muséum National d'Histoire Naturelle, CNRS, SU Paris France; ^2^ Institut de Systématique, Evolution, Biodiversité (ISYEB) UMR 7205, Muséum National d'Histoire Naturelle, CNRS, SU, EPHE‐PSL, UA Paris France

**Keywords:** bony labyrinth, burrowing, ecological niches, geometric morphometrics, semicircular canals, *Talpa*

## Abstract

The inner ear, particularly the semicircular canals system, plays a crucial role in balance and spatial navigation. It has previously been investigated to understand if its shape is related to the ecology: it is indeed known to be a good predictor for the extreme ecological niche of burrowing or arboreal organisms, although being strongly driven by the phylogeny. Recent phylogenetic studies of European moles have revealed the paraphyletic status of the species *Talpa europaea* and the description of new parapatric species in south‐western Europe. Following the description of the new mole species *Talpa aquitania*, its forelimb morphology has been compared to those of its two sister species, *Talpa europaea* and *Talpa occidentalis*, revealing inter‐ and intra‐specific morphological discrimination. The present study aims to compare the morphologies of the semicircular canal system in these three sister species of moles. Geometric morphometrics was used on 58 specimens representing the three species to analyze shape‐related information and quantify the variability. The results demonstrate interspecific variability in the shape of the semicircular canals within the three species and intraspecific variability across sampling sites. Furthermore, no sexual dimorphism was observed for the bony labyrinth. The observed variability is likely influenced by the distinct ecological characteristics of the habitats housing the three *Talpa* species and their populations or by genetic differences resulting from their evolutionary history.

## Graphical abstract

The study investigates the shape of the semicircular canals in the inner ear of three sister mole species—*Talpa europaea*, *Talpa aquitania*, and *Talpa occidentalis*—using geometric morphometrics on 58 specimens. It finds both interspecific and intraspecific variability in canal morphology, but no evidence of sexual dimorphism. This variation is likely influenced by ecological factors and genetic divergence shaped by evolutionary history.

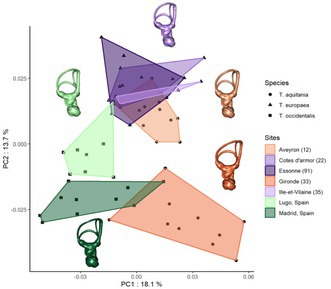

## 1 INTRODUCTION

In 1758, Linnaeus introduced the genus *Talpa*, which was initially placed within the traditional order of “insectivores”, a polyphyletic gathering of Laurasian placentals, such as hedgehogs, shrews, and moles, now reclassified under Eulipotyphla (Waddell et al., 1999). The European mole, *Talpa europaea* Linnaeus, 1758, has historically been recognized as a single species. However, recent molecular analyses utilizing mitochondrial and nuclear genes have revealed that it is not monophyletic (Feuda et al., 2015; Nicolas et al., 2017a). Consequently, a new species, *Talpa aquitania*, has been described by Nicolas et al. (2017b) and identified as the sister taxa to *Talpa occidentalis* Cabrera, 1907. These three species are delimited by molecular studies and several morphological characters, including eyelid fusion and the shape of the mesostyles of the first, second, and third upper molars (Nicolas et al., 2017a).

These three species co‐occur in south‐western Europe where they have allopatric or parapatric distributions: *T. occidentalis* is endemic to the Iberian Peninsula, *T. aquitania* is present in Northern Spain and south‐western France, and *T. europaea* is widely distributed from France (North and South‐eastern in majority) to Russia (Nicolas et al., 2017a; Wilson & Mittermeier, 2018). *T. occidentalis* and *T. aquitania* co‐occur in Northern Spain. *T. europaea* and *T. aquitania* have mostly allopatric geographical distributions on either side of the Loire River, although the latter does not represent a strict barrier between the two species, as small areas of contact exist in the Pyrénées mountains and the Var department (Nicolas et al., 2021). The factors explaining this geographical distribution remain unknown, but several hypotheses based on historical processes, contemporary environmental variables, and species interactions were proposed (Nicolas et al., 2017b, 2021).

A recent morpho‐functional study based on the forelimbs of these three *Talpa* species highlighted differences in the morphology of the ulna and humerus, which may impact their digging performance (Costes et al., 2023). It has been proposed that such variation may be due to the properties of the different soils in which moles dig, such as their compaction, although it is only a hypothesis that still needs to be investigated. To further investigate this hypothesis, we will examine the osseous inner ear of the exact same specimens studied by Costes et al. (2023).

**FIGURE 1:**
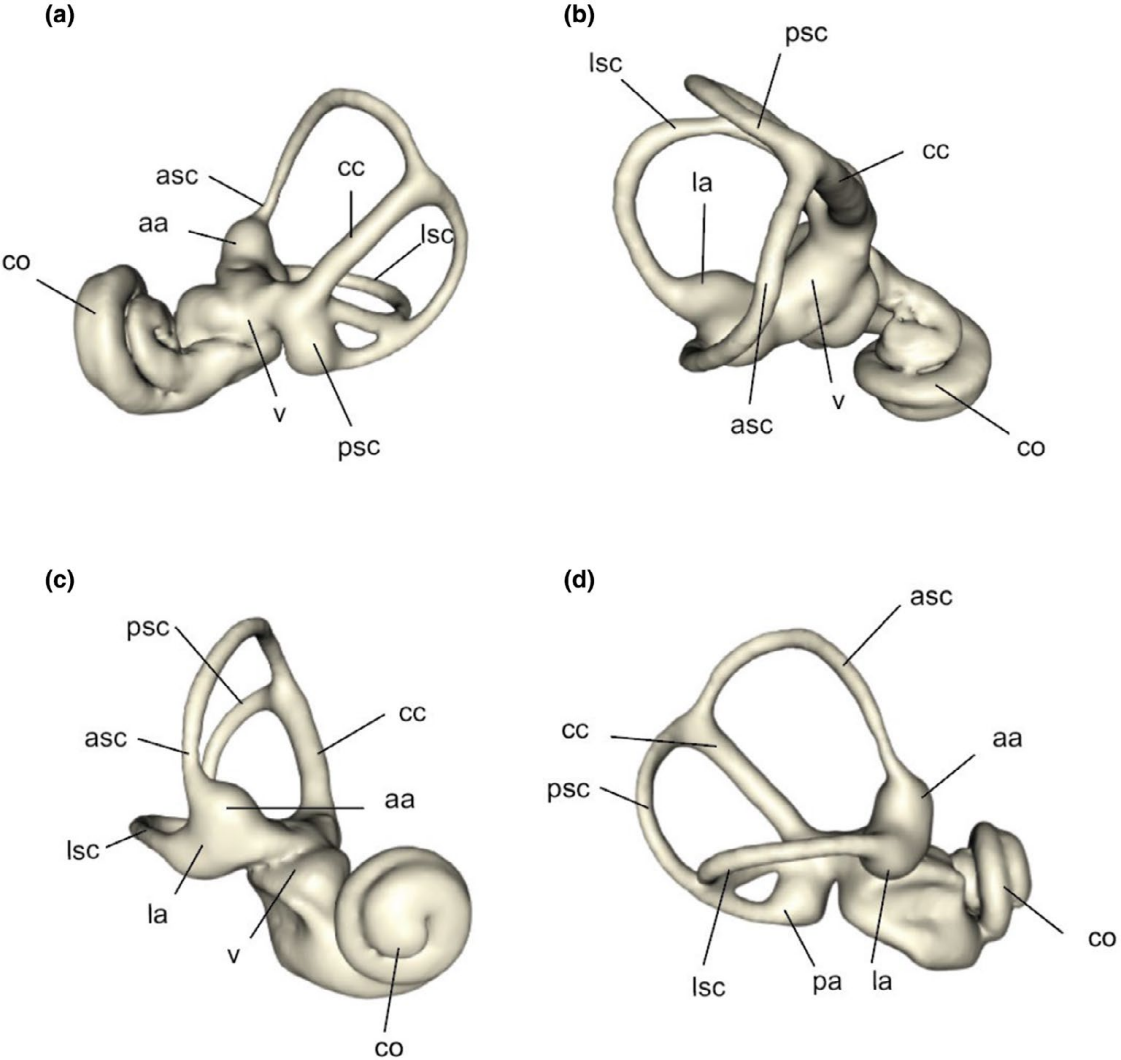
3D model of the bony labyrinth of the inner ear of the specimen MNHN‐ZM‐2018‐2242 (*Talpa aquitania*), considered as the closest specimen to the estimated mean shape. The ears are positioned in anatomical views: Posterior (a), dorsal (b), anterior (c), and lateral (d) views. aa, anterior ampulla; asc, anterior semicircular canal; cc, common crus; co, cochlea; la, lateral ampulla; lsc, lateral semicircular canal; pa, posterior ampulla; psc, posterior semicircular canal; v, vestibule. Not to scale.

The bony labyrinth of the inner ear is a complex system of interconnected spaces within the petrosal bone, containing perilymph that suspends the membranous labyrinth. It can be divided into two main regions: the pars cochlearis, which houses the cochlea and vestibule, and the pars canalicularis, which encloses the semicircular canals, ampullae, and the common crus. The cochlear duct within the membranous labyrinth contains the spiral organ for hearing, while the saccule and utricle in the vestibule house receptors sensitive to linear motion. The superior portion of the membranous labyrinth detects rotational head movements through the semicircular ducts, ampullae, and their connections via the common crus between the anterior and posterior ducts (Blanks et al., 1975; Spoor, 2003). Each side of the skull contains three semicircular canals—anterior, posterior, and lateral— each containing its respective semicircular duct (Figure 1). The anterior and posterior canals, considered vertical, converge at the common crus, while the lateral canal, oriented horizontally, connects in the area of the posterior ampulla. These canals are generally orthogonal on the same side (Berlin et al., 2013) and feature ampullae with crista ampullaris, specialized structures that detect head movement.

The semicircular canals play a vital role in maintaining balance and spatial navigation by interpreting the direction, speed, and amplitude of head movement. Hair cell activation within one canal is coupled with inhibition in its contralateral counterpart due to their orientation (Coutier et al., 2017). This system provides critical input for reflexes such as the vestibulo‐colic reflex, which stabilizes head posture, and the vestibulo‐ocular reflex, which stabilizes gaze during movement (Highstein et al., 2004). Together, these mechanisms ensure proper balance, spatial orientation, and navigation.

Previous studies have linked the morphology of the bony labyrinth and semicircular canals to locomotion speed (e.g., Billet et al., 2012; Grohé et al., 2018), fossorial lifestyle (e.g., Maddin & Sherratt, 2014), head motion, and locomotion linked to agility (e.g., Malinzak et al., 2012; Spoor et al., 2007), and domestication (Evin et al., 2022). Several studies also have highlighted the impact of phylogeny on the morphology of semicircular canals (e.g., Latimer et al., 2023). The crucial role of the semicircular duct system in balance and spatial navigation makes this organ, and the bony cavity that contains it, a potential indicator of the digging behavior in species of interest.

The aim of this study is to quantify, through 3D geometric morphometric methods, the interspecific variability of the semicircular canals morphology in order to determine if and how the three *Talpa* species differ in their digging capabilities and to confirm the previous hypotheses regarding differences in digging forces, as highlighted by Costes et al. (2023). This study will also test the hypothesis of a sexual dimorphism and quantify the intraspecific variability of the semicircular canals morphology. As we only study three species, the impact of phylogeny will not be considered.

## 2 MATERIALS AND METHODS

### 2.1 Sample

A total of 58 bony labyrinths from the three species of *Talpa* have been analyzed. All the specimens come from the collections of the Muséum national d'Histoire naturelle at Paris (MNHN) for *T. aquitania* and *T. europaea*, and from the Donana Biological Station (Seville, Spain) for *T. occidentalis* (Table 1; Supporting Information S1). Both sexes are represented, and only the adults have been considered. The specimens are from 16 different sites (Table 1). Sites from the same French departments and Spanish provinces are grouped in this study to facilitate the understanding of the graphics. The selected sites are two Spanish provinces: Lugo and Madrid, and five French departments: Aveyron, Gironde, Côtes d'Armor, Essonne, and Ille‐et‐Vilaine.

**TABLE 1:** Number of specimens depending on the species, the sexes, and the sampling sites.

Species	Number of specimens	Sex	Sites
*Talpa europaea*	20	Male: 7 Female: 13	Essonne: 10
			Ille et Vilaine: 4
			Côtes d'Armor: 6
*Talpa aquitania*	20	Male: 10 Female: 10	Gironde: 10
			Aveyron: 10
*Talpa occidentalis*	18	Male: 9 Female: 9	Lugo: 8
			Madrid: 10

All specimens underwent X‐ray microtomography scanning. Specimens from the MNHN collections were scanned with a resolution of 31 μm at the AST‐RX platform (UAR 2 AD) of MNHN, using the “v|tome|x L 240‐180” device from Baker Hughes Digital Solutions. Specimens from the Spanish collections were scanned with a resolution of 31.99 μm at the Microtomography Platform of the University of Poitiers (IC2MP—UMR 7285), using the “EasyTom XL Duo tube microfocus 150kV” device from RX Solutions. Manual segmentation of the semicircular canals was performed using Mimics 21.0 ©Materialise software at the 3D Imaging Workshop of CR2P. The 3D models were then exported as stl files.

### 2.2 Study of morphological variations using 3D geometric morphometrics

#### 2.2.1 Landmarks

**FIGURE 2:**
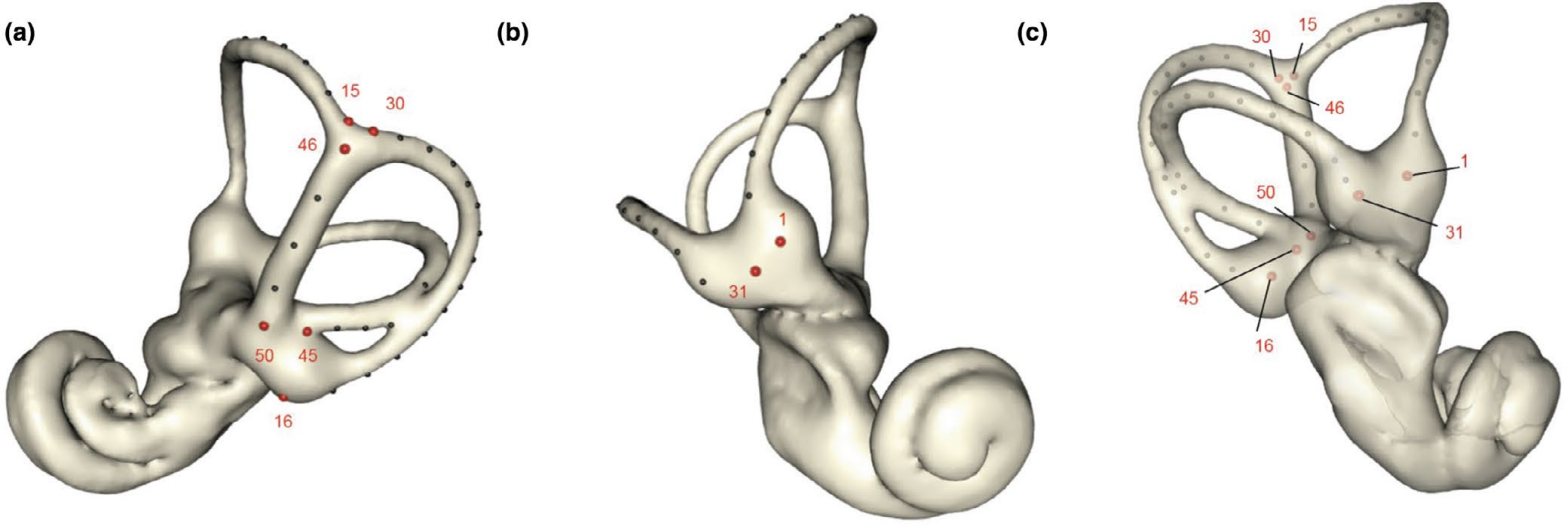
3D model of the bony labyrinth of the inner ear of the specimen MNHN‐ZM‐2018‐2242 (*Talpa aquitania*), here considered as the closest specimen to the estimated mean shape. The modeling and visualization of externally placed landmarks were performed using R version 4.2.2 (R Core Team, 2022) with the *rgl* package (Adler et al., 2019) and the associated functions *shape3d* and *plot3d*. (a): Posterior view. (b): Anterior view. (c): View showing the internal landmarks. The red points represent fixed landmarks, and the black points represent sliding landmarks.

We used 3D geometric morphometrics to analyze the shape variation of the semicircular canals between the three *Talpa* species, which is a statistical analysis of landmark‐based shape variation (Zelditch et al., 2012). The landmarking process was carried out using the Aviso 3D 2021.1 software. The protocol (Supporting Information S2) is adapted from a protocol originally applied to the membranous labyrinth (David et al., 2016). One set of landmarks was placed on the mid‐section of the semicircular canals and common crus, using Aviso's skeletonization tool. Another set of landmarks was placed on the external surface of the semicircular canals and the common crus. To cover the entire length of the canals, landmarks were placed from the center of the ampulla, at the level of the crista, to the end of each semicircular canal (i.e., reaching the vestibule or common crus). To cover the length of the common crus, landmarks are placed from the most dorsal to the most ventral part of the common crus. Fifteen landmarks are positioned for the external part of each semicircular canal (including two fixed and thirteen sliding). For the common crus, points are placed from the junction of the anterior and posterior canal to the vestibule (two fixed and three sliding). The same number is placed in the same manner for the internal part. Eight datasets per individual are recorded: two for the common crus (internal and external) and two for each of the semicircular canals (Figure 2).

A total of seven independent curves are therefore defined on the semicircular canals, each of which starts with a homologous landmark (Type I). The eight Type I (fixed) landmarks are positioned at the beginning of each curve, according to a detailed protocol, referencing specific anatomical points (Supporting Information S2). The sliding landmarks are arbitrarily placed along curves as they can slide while minimizing the bending energy. Our definition of the homologous landmarks allows access to the angles, lengths, thicknesses, and twists of the semicircular canals, as well as the length of the crus commune. The raw material (meshes and landmarks) is available on request.

#### 2.2.2 Statistical analyses

Statistical analyses were performed using R software version 4.2.2 (R Core Team, 2022; Supporting Information S3). A generalized procrustes analysis was conducted using the *gpagen* function from the *geomorph* package (Adams et al., 2021). Generalized Procrustes Analysis (Gower, 1975) translates all specimens, scales them to the same size, and optimally rotates them, using a least squares criterion, until the coordinates of homologous points align as closely as possible. It includes both fixed and sliding landmarks, which slide during the analysis. To visualize shape differences among specimens, a Principal Component Analysis (PCA) was performed using the *gm.prcomp* function from the *geomorph* package (Adams et al., 2021).

We measured the angles between the anterior and posterior semicircular canals, as well as between the lateral and posterior semicircular canals for each species morphological consensus (i.e., the mean shape estimate) using ImageJ.

We performed a linear discriminant analysis (LDA) using the Procrustes coordinates as the dependent variable, with size, species, sex, and sampling sites as predictors, to assess how effectively the specimens could be discriminated based on these variables. The LDA allowed us to determine the contribution of each predictor to the separation of the groups, providing insight into how size, species, and sampling site influence the observed morphological variation. To perform this LDA, we used the *mvols* and *mvgls.dfa* functions from the *MvMORPH* package (Clavel et al., 2015).

To assess the significance of the different predictors, a multivariate analysis of variance (MANOVA) was conducted using the *manova.gls* function from the *mvMORPH* package (Clavel et al., 2015). This approach allowed us to evaluate the contribution of each predictor (size, species, sex, and sampling site) to the variation in the response variable (Procrustes coordinates), considering the multivariate nature of the data. In addition to the global MANOVA test, post‐hoc pairwise comparisons were performed, using the *pairwise.glh* function from the *mvMORPH* package, to identify which sampling site predictors were significantly different from one another. This combined approach provided a comprehensive understanding of how each predictor and their levels influenced the morphological differentiation among the specimens.

In geometric morphometrics, the effect of allometry (i.e. the size‐related modifications of morphological features (Klingenberg, 2016)) is considered a recurring factor explaining shape variation (Mitteroecker et al., 2013). Here, we focused on static or size allometry (i.e., the allometry observed at a single ontogenetic stage), which was studied by including the size variable in our linear discriminant analysis and the MANOVA. This allowed us to control for the effect of body size on morphological variation, ensuring that shape differences among the specimens were not confounded by size‐related changes. Neither ontogenetic allometry (i.e., the association between size and shape across different ontogenetic stages) nor evolutionary allometry (i.e., the size change induced by evolutionary processes and species dependent) was considered in this study.

## 3 RESULTS

**FIGURE 3:**
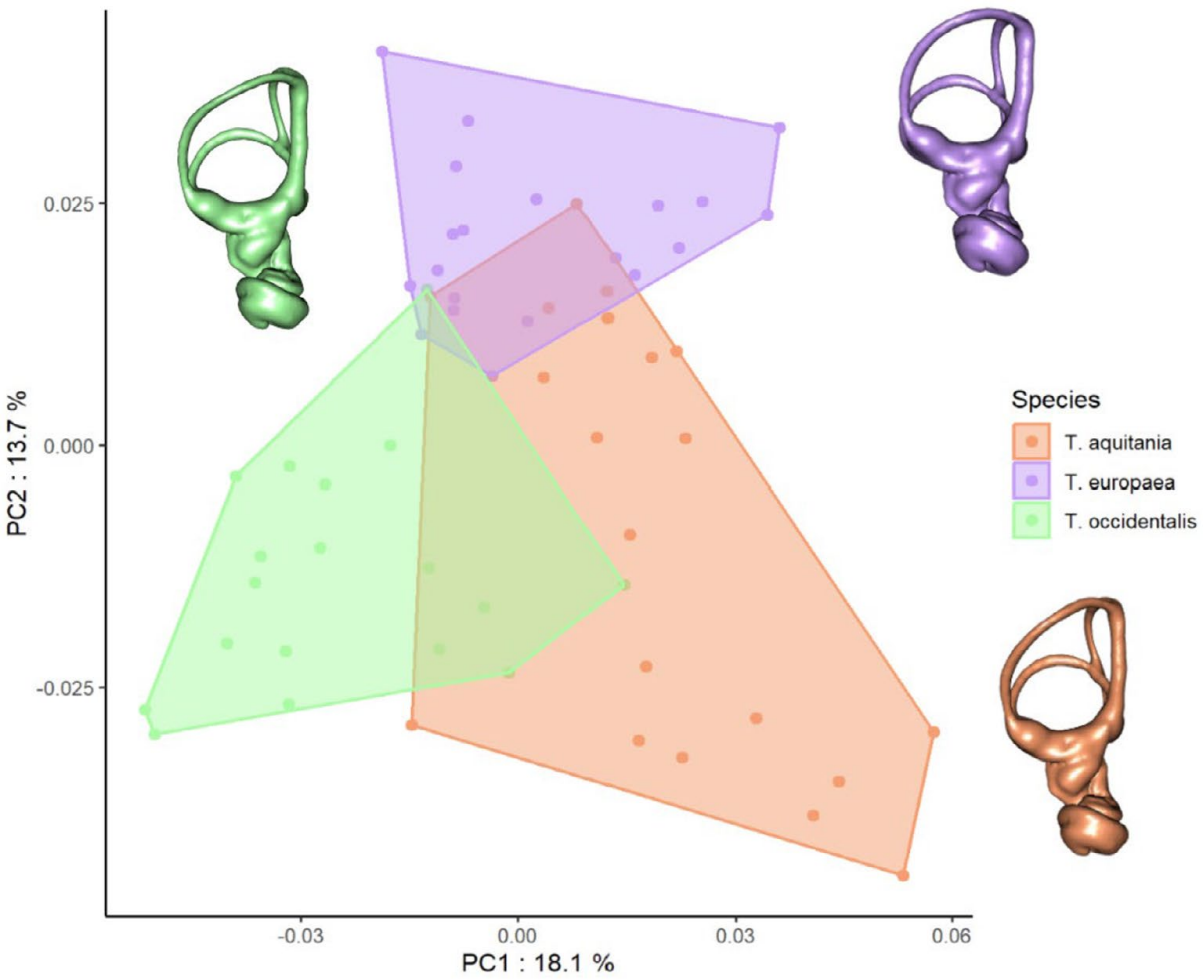
Results of the PCA performed on the morphometric data of the bony labyrinth. The different species are represented by points and convex hulls of different colors. The morphological consensus of the bony labyrinth per species is represented by a 3D mesh of the same view. The three species are differentiated with a small overlap.

The first four PCs account for 51.46% of the overall variance in the semicircular canals shape, and the first two PCs account for 31.8%. The visualization of the morphological spaces in the first two axes enables discrimination of three groups corresponding to the three *Talpa* species (Figure 3).

The morphological consensus per species (Figure 3) reveals several differences in the semicircular canal conformation. The anterior semicircular canal and the crus commune are circumscribed in a narrower area and display a greater ellipticity for *Talpa occidentalis*. The crus commune of *Talpa aquitania* is almost as long as that of *Talpa occidentalis*, but the area between the crus commune and the anterior semicircular canal is wider and less elliptical. Finally, Talpa europaea has generally more rounded semicircular canals, with the three canals and crus commune being thicker than in the other species.

**FIGURE 4:**
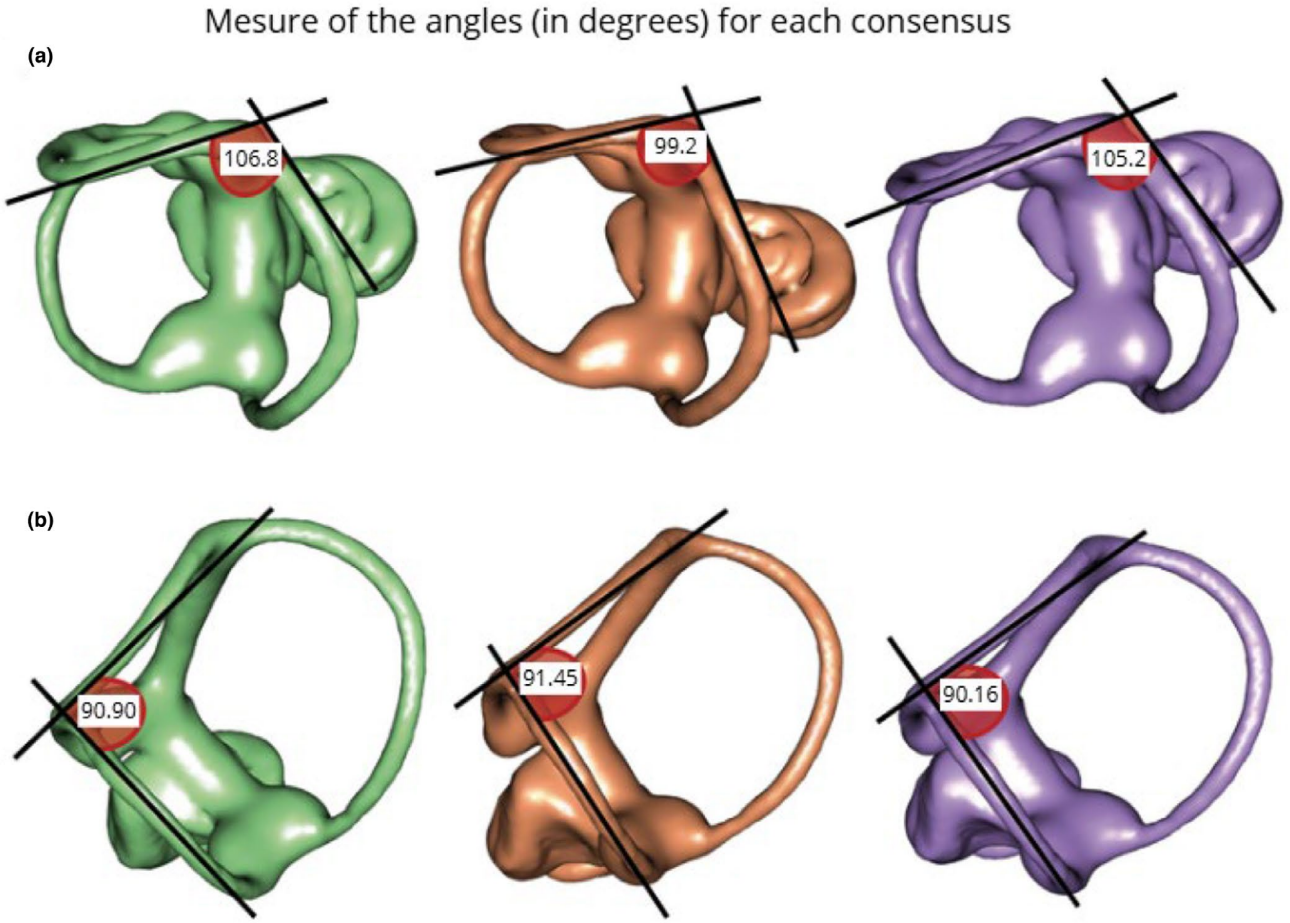
Distance to the orthogonality of the semicircular canals. The measurements were made with ImageJ, on the mean shapes estimated for each species. (a) Anterior and posterior semicircular canals. (b) Lateral and posterior semicircular canals. green: *Talpa occidentalis*, orange: *Talpa aquitania*, purple: *Talpa europaea*.

The orthogonality of the semicircular canals refers to each canal lying in a plane and crossing the planes of the other two canals at 90°. The nearest to orthogonality between the anterior and posterior semicircular canals is *T. aquitania*, and the least near is *T*. *occidentalis* (Figure 4a). The distance to orthogonality between the lateral and posterior semicircular canals is almost similar between the three species, the nearest being *T. europaea*, and the least near being *T. aquitania* (Figure 4b).

**FIGURE 5:**
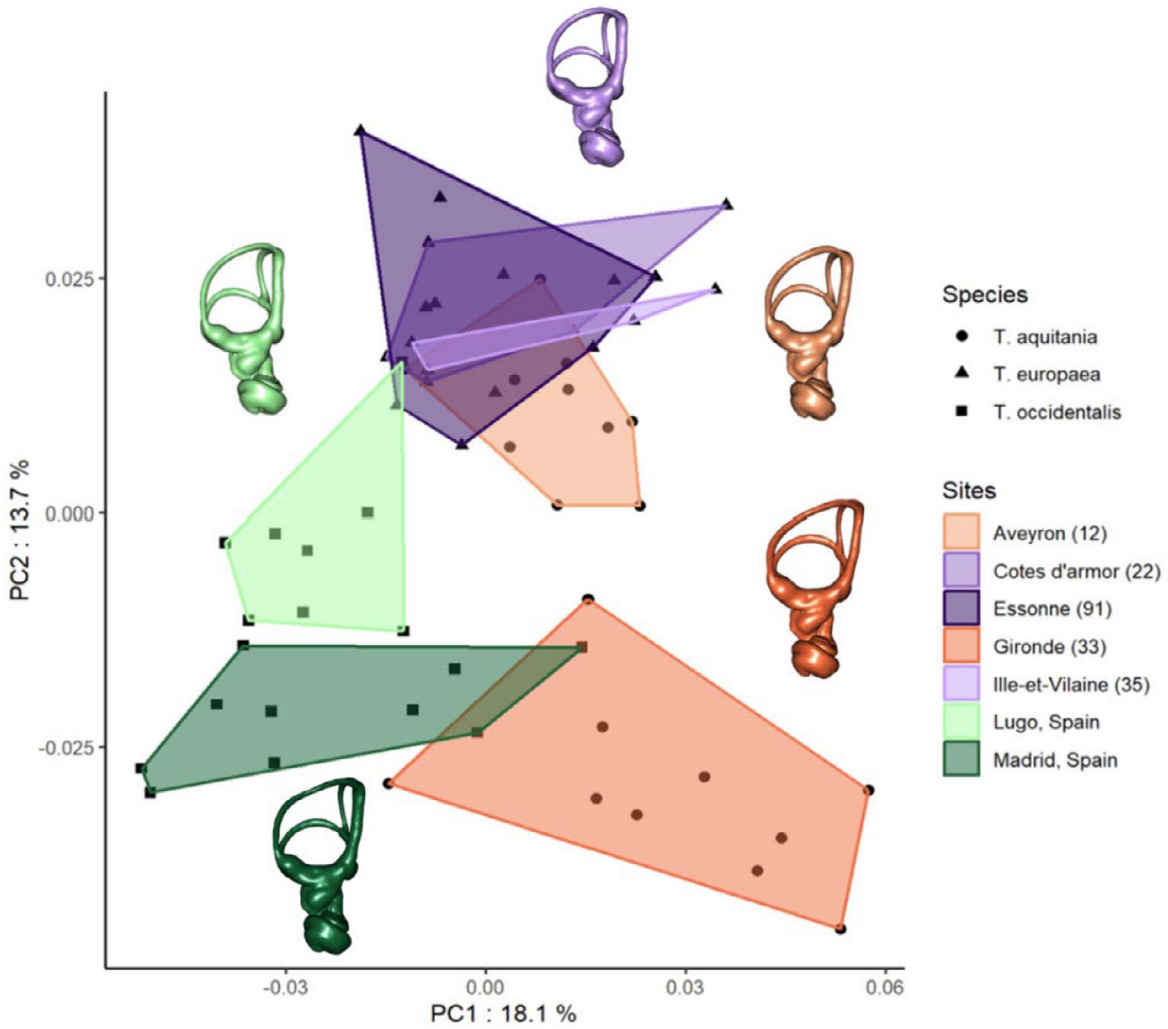
Results of the PCA conducted performed on the morphometric data of the bony labyrinth. The different sites are visualized by convex hulls of different colors, and the different species are represented by points of different shapes. *Talpa europaea*, purple triangle; *Talpa aquitania*, orange circle; *Talpa occidentalis*, green square. The morphological consensus of bony labyrinth per sampling sites (*T. aquitania* and *T. occidentalis*) and for all sampling sites (*T. europaea*) is represented by a 3D mesh of the same view. There is a clear differentiation between sampling sites for *T. aquitania* and *T. occidentalis* but none for *T. europaea*.

The PC1 and PC2 show a clear discrimination of the effect of geographic sites in the morphological variation of the semicircular canals (Figure 5). The PC1 discriminates the Spanish sites from the sites in Gironde and Aveyron. The PC2 shows a clear differentiation between the two Spanish sites from which *T. occidentalis* originates and a good differentiation between Aveyron and Gironde, from which *T. aquitania* originates. The sites corresponding to the species *T. europaea* are not differentiated by any axes and overlap with the Aveyron site, from which *T. aquitania* originates (Figure 5). The morphological consensus per sampling sites clearly shows morphological differences inside the species *T. aquitania* and *T. occidentalis* and between different sampling sites. *T. aquitania* specimens from Aveyron, which are more similar to *T. europaea* specimens, have a narrower area and greater ellipticity between the crus commune and the anterior semicircular and thicker semicircular canals than specimens from Gironde (Figure 5). The same differences are shown for the Lugo and Madrid specimens of *Talpa occidentalis*, where the Lugo specimens exhibit a narrower area and greater ellipticity between the crus commune and ASC together with narrower semicircular canals.

The distribution of the morphological variation in the different PCs does not show any clear differences related to the sex of the individuals (Supporting Information S4).

**FIGURE 6:**
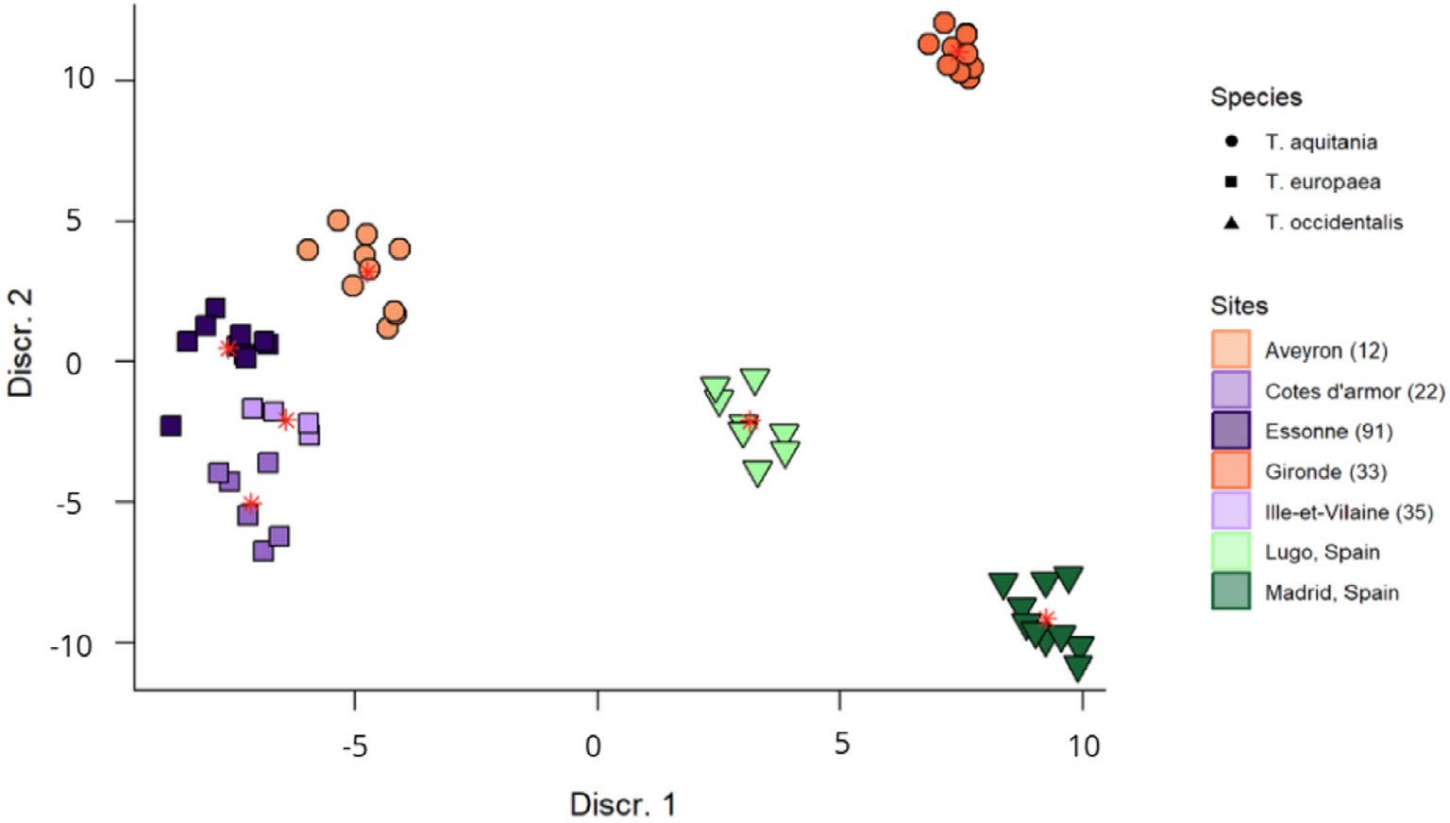
Linear discriminant analysis of *Talpa* species with geographical site differentiation. The three *Talpa* species and their respective sites are distinct in the discriminant space, except for *T. europaea* for which the three geographic sites are barely distinctive. The centroids for each species are represented by a red star.

The linear discriminant analysis (Figure 6) identified six discriminant axes in total. The first two discriminant axes accounted for a substantial portion of the total variation (62%), indicating that these two axes capture most of the differences between the groups. The LDA shows clear patterns of morphological separation among the three *Talpa* species (*T. aquitania*, *T. europaea*, and *T. occidentalis*) as well as sampling site differentiation, except for the three sites of *T. europaea*, which are barely separated in the discriminant space. This is not surprising for the Côtes d'Armor and Ille‐et‐Vilaine sampling sites, as they are only separated by seven kilometers. However, Essone, which is geographically far from the two Breton sites, is also not discriminated. The centroids (red stars) for each species cluster confirm the separation of the sampling sites for *T. aquitania* and *T. occidentalis* in the discriminant space. Meanwhile, the centroids for *T. europaea* sampling sites are closer, indicating greater similarity or shared traits within this species.

The MANOVA confirms that sampling sites and species had a statistically significant effect on the morphological variation (*p* = 0.001) indicating clear morphological differentiation for these factors. In contrast, size and sex did not show significant effects on morphology (*p* = 1.000 for size and *p* = 0.188 for sex), suggesting that these factors do not significantly contribute to shape variation among the individuals sampled.

No significant morphological differences were observed between the different sampling sites of *Talpa europaea* (adjusted *p* = 0.29963 between Côtes d'Armor and Essonne, 0.86392 between Côtes d'Armor and Ille‐et‐Villaine, 0.86392 between Essonne and Ille‐et‐Villaine).

Within *Talpa occidentalis*, located in Lugo and Madrid, significant differentiation was found between the two sites (adjusted *p* = 0.02622). The same difference was found between the two sites, Gironde and Aveyron, of *Talpa aquitania* (adjusted ppp = 0.02622).

## 4 DISCUSSION

One of the most striking adaptations to the extreme confinement to the underground dark environment is certainly the vision loss, which occurred in many subterranean mammals, together with the acquisition of compensatory traits for blindness (Nevo, 1999). Consequently, moles are expected to exhibit enhanced spatial navigation and detection of body motion and orientation. These adaptations include an increase in sensitive skin, vibrissae, and tactile hairs on the head and body, facilitating perception of the underground environment and a unique tactile orienting behavior (e.g., Begall et al., 2007; Catania & Remple, 2003; Crish et al., 2003; Partha et al., 2017). Additionally, moles possess a finely tuned perception of the Earth's magnetic field, aiding in their unique directional orientation (Kimchi & Terkel, 2001; Marhold et al., 1997; Nevo, 1999).

In our study, we found that the three *Talpa* species exhibit distinct morphological variations in their semicircular canals, which likely reflect adaptations to head motion during digging and navigation through different substrates. Morphological variations such as ellipticity, thickness, orthogonality, orientation, and curvature of semicircular canals are here scrutinized to discuss whether they are involved in digging functions. The sensitivity of the semicircular canals is influenced by their size and shape (Muller, 1994). The semicircular canals of *Talpa occidentalis* are thinner compared to those of the two other *Talpa* species, indicating their reduced sensitivity (Goyens et al., 2022). Moreover, they exhibit a greater ellipticity, potentially also resulting in their reduced sensitivity, as observed in squamates (Goyens, 2019; but see McVean, 1999 on talpids). Therefore, *Talpa occidentalis* might engage its head more during locomotion than the other species, or its digging activity might be made more challenging by the characteristics of the soil, potentially explaining this reduced sensitivity compared to the other two species. It is usual to find that subterranean organisms have greater sensitivity (Goyens et al., 2022; Pfaff et al., 2015), except for those that involve the head in their digging activity and have a reduced visual system (Maddin & Sherratt, 2014). This reduced sensitivity may allow for angular head movements with a higher angular velocity, preventing saturation of the semicircular canals with excessive information (David et al., 2016). Also, the semicircular canals of *Talpa occidentalis* are the least near to orthogonality canals, which can indicate a lower mean vestibular sensitivity. The more the angle between the planes of the semicircular canals deviates from 90°, the lower the average vestibular sensitivity, with burrowing species showing twice as much deviation as non‐burrowers (Berlin et al., 2013). Species with a greater deviation from orthogonality tend to have slower head locomotor movements and lower mean vestibular sensitivity (Berlin et al., 2013; Billet et al., 2012).

Here, we were able to discriminate among the three *Talpa* species based on their semicircular canal morphologies, consistent with the findings of Costes et al. (2023) in their study of forelimb morphology. The few number of morphological differences between the three species may reflect their short evolutionary history with a divergence time dated back to the Pliocene– Pleistocene boundary (Feuda et al., 2015; Nicolas et al., 2017b). The similarity between the morphological consensus of *T. occidentalis* and *T. aquitania* is not surprising as they share repeated DNA sequences and are sister species (Aleix‐Mata et al., 2020), which diverged from a common ancestor with *T. europaea*. The three species do not show sexual dimorphism in the shape of the bony labyrinth of their inner ear. Apart from humans (Braga et al., 2019) and the wild boar (Evin et al., 2022), no sexual dimorphism has ever been shown in other mammals for this anatomical structure.

Furthermore, significant variations in semicircular canal morphologies were observed among individuals captured at different sites, especially for *T. aquitania* and *T. occidentalis* (Figure 5). Two morphological groups had already been highlighted for *T. aquitania* based on the ulna and, to a lesser extent, on the humerus (Costes et al., 2023) but not for *T. occidentalis*. The intraspecific variation in the morphology of the inner ear has already been demonstrated in populations of artiodactyls and the *Homo* genus, for example (Mennecart et al., 2022; Ponce de León et al., 2018). This intraspecific variation based on geography points to potential local adaptations, phenotypic plasticity depending on environmental conditions, or genetic structuring within species. Phylogenetic analysis of the Cytochrome b gene revealed the presence of 4 sublineages within *T. aquitania* (Nicolas et al., 2017a). Two of these sublineages, with an estimated divergence time of 0.349 ± 0.050 My, correspond to all specimens from Gironde on one hand and all specimens from Aveyron included in this study on the other. This genetic structuring is probably explained by a history of population allopatric differentiation in multiple refugia during the Pleistocene. It could have allowed morphological differentiation between these populations either by drift or local adaptation. It is interesting to note that the Gironde and Aveyron populations were probably captured in different soil types, which could also have contributed to potential morphological differentiation between the two populations through phenotypic plasticity or adaptation. The Aveyron population of *T. aquitania* is really close to the morphological spaces occupied by *T. europaea* in our statistical analyses. This might be explained by the geographical proximity between them and some *T. europaea* populations of Southern France (e.g., map of distribution in Nicolas et al., 2021), which might have led to a genetic admixture, although this hypothesis remains unproven and not very plausible. On the contrary, the Gironde population is far away from any *T. europaea* population, which can explain the distance between them in the morphological spaces. No significant variations in semicircular canal morphologies were observed among *T. europaea*, even between Essonne and the two other sampling sites in Brittany, which are far from each other. This could be explained by both the absence of genetic structure between these populations (Nicolas et al., 2017a) and similar soil types. Unfortunately, no data are available on the genetic divergence between the Lugo and Madrid populations, but some phylogeographic structure has been observed within *T. occidentalis*.

It is noteworthy that our study focused solely on the semicircular canals, deliberately omitting analysis of the vestibule and cochlea. The cochlea, as the organ of hearing, is often evaluated based on its elongation and coiling, which serve as proxies for hearing capacities (Manoussaki et al., 2008; West, 1985). Future research would benefit from a complementary analysis of cochlear shape, which could provide valuable insight into the ability to detect sounds within a specific frequency range and its adaptation to its environment (e.g., Costeur et al., 2018; Del Rio et al., 2023).

An interesting perspective would be to scrutinize the membranous labyrinth to estimate functional parameters, using the method of David et al. (2016). It would give a unique view of the mechanics of the head movements, with, for instance, the “mechanical sensitivity” that reflects the biomechanical response of the semicircular canals to head movement and has been linked to the burrowing lifestyle in a previous study (Selva, 2017).

## 5 CONCLUSION

Our study highlighted interspecific anatomical differences of the semicircular canals of the inner ear among *T. europaea*, *T. aquitania*, and *T. occidentalis*. These modifications particularly involve the shape of the anterior semicircular canal and the relative thickness of the canals, and may result in a difference in vestibular sensitivity. This could potentially be explained by the different constraints imposed by their respective environments, such as soil compactness or plasticity, composition (e.g., clay, sand), colonization by roots, or subtle genetic divergences due to their evolutionary history, which have been driven by climatic oscillations during the Quaternary. Intraspecific morphological differences are also observed within each species, with two distinct morphological groups corresponding to the two sampling sites identified for *T. aquitania* and *T. occidentalis*. No sexual dimorphism is observed at either the interspecific or intraspecific level. To better understand the evolutionary context and selection pressures on these species and the genus *Talpa* as a whole, ecological data, precise descriptions of their habitat, and a larger study including the impact of the phylogeny are needed. To further understand the biomechanical response of the semicircular canals to the environmental constraints to which each species can be subjected, a comprehensive analysis of the membranous labyrinth of the inner ear would be a good path for future research.

## AUTHOR CONTRIBUTIONS

AM carried out the acquisition of morphological data, performed the statistical analyses, interpreted the data, and drafted the manuscript. SL and VN conceived and designed the study, collecting data. NP‐C segmented the bony inner ears of the study. SDT contributed to statistical analyses and interpretation of data. AM, SDT, and SL have equally contributed to this article. All authors revised and approved the manuscript.

## CONFLICT OF INTEREST STATEMENT

The authors declare that they have no conflict of interest.

## Supporting information


Supporting Information S1.

Supporting Information S2.

Supporting Information S3.

Supporting Information S4.

## Data Availability

The data that support the findings of this study are available from the corresponding author upon reasonable request.
