# Supplementary material for: Morphometric study of the bony labyrinth of the inner ear in the European moles Talpa europaea, Talpa occidentalis, and Talpa aquitania
Source: J Anat. 2025 Jul 3;248(1):71–81. doi: 10.1111/joa.70017 (PMC12682593; doi:10.1111/joa.70017)
Supplement: Supplementary file 1 — Supporting Information S1. [file JOA-248-71-s002.docx]

**SUPPORTING INFORMATION 1: Table summarizing the data (collection number, species, locality, sex) of the individuals of the sampling**

| **#_specimen** | ***species_name*** | **locality** | **sex** |
| --- | --- | --- | --- |
| MNHN-ZM-2017-2254 | *T. aquitania* | Gironde (33) | M |
| MNHN-ZM-2017-2257 | *T. aquitania* | Gironde (33) | M |
| MNHN-ZM-2017-2258 | *T. aquitania* | Gironde (33) | F |
| MNHN-ZM-2017-2259 | *T. aquitania* | Gironde (33) | M |
| MNHN-ZM-2017-2260 | *T. aquitania* | Gironde (33) | F |
| MNHN-ZM-2017-2261 | *T. aquitania* | Gironde (33) | M |
| MNHN-ZM-2017-2271 | *T. aquitania* | Gironde (33) | M |
| MNHN-ZM-2017-2273 | *T. aquitania* | Gironde (33) | F |
| MNHN-ZM-2017-2274 | *T. aquitania* | Gironde (33) | F |
| MNHN-ZM-2017-2275 | *T. aquitania* | Gironde (33) | F |
| MNHN-ZM-2018-2239 | *T. aquitania* | Aveyron (12) | F |
| MNHN-ZM-2018-2240 | *T. aquitania* | Aveyron (12) | M |
| MNHN-ZM-2018-2241 | *T. aquitania* | Aveyron (12) | M |
| MNHN-ZM-2018-2242 | *T. aquitania* | Aveyron (12) | F |
| MNHN-ZM-2018-2243 | *T. aquitania* | Aveyron (12) | F |
| MNHN-ZM-2018-2244 | *T. aquitania* | Aveyron (12) | M |
| MNHN-ZM-2018-2245 | *T. aquitania* | Aveyron (12) | F |
| MNHN-ZM-2018-2246 | *T. aquitania* | Aveyron (12) | F |
| MNHN-ZM-2018-2247 | *T. aquitania* | Aveyron (12) | M |
| MNHN-ZM-2018-2248 | *T. aquitania* | Aveyron (12) | M |
| MNHN-ZM-1983-777 | *T. europaea* | Essonne (91) | M |
| MNHN-ZM-1983-778 | *T. europaea* | Essonne (91) | F |
| MNHN-ZM-1993-1708 | *T. europaea* | Essonne (91) | F |
| MNHN-ZM-1993-3218 | *T. europaea* | Essonne (91) | M |
| MNHN-ZM-1993-3219 | *T. europaea* | Essonne (91) | M |
| MNHN-ZM-1993-3220 | *T. europaea* | Essonne (91) | M |
| MNHN-ZM-1993-3221 | *T. europaea* | Essonne (91) | F |
| MNHN-ZM-1993-3222 | *T. europaea* | Essonne (91) | F |
| MNHN-ZM-1993-3234 | *T. europaea* | Essonne (91) | M |
| MNHN-ZM-1993-3235 | *T. europaea* | Essonne (91) | F |
| MNHN-ZM-2018-594 | *T. europaea* | Cotes d'armor (22) | M |
| MNHN-ZM-2018-595 | *T. europaea* | Cotes d'armor (22) | F |
| MNHN-ZM-2018-596 | *T. europaea* | Ille-et-Vilaine (35) | F |
| MNHN-ZM-2018-598 | *T. europaea* | Cotes d'armor (22) | M |
| MNHN-ZM-2018-599 | *T. europaea* | Cotes d'armor (22) | F |
| MNHN-ZM-2018-600 | *T. europaea* | Ille-et-Vilaine (35) | F |
| MNHN-ZM-2018-603 | *T. europaea* | Cotes d'armor (22) | F |
| MNHN-ZM-2018-604 | *T. europaea* | Cotes d'armor (22) | F |
| MNHN-ZM-2018-605 | *T. europaea* | Ille-et-Vilaine (35) | F |
| MNHN-ZM-2018-606 | *T. europaea* | Ille-et-Vilaine (35) | F |
| 18074 | *T. occidentalis* | Lugo, Spain | F |
| 18082 | *T. occidentalis* | Lugo, Spain | F |
| 18086 | *T. occidentalis* | Lugo, Spain | M |
| 18087 | *T. occidentalis* | Lugo, Spain | M |
| 18081 | *T. occidentalis* | Lugo, Spain | M |
| 18088 | *T. occidentalis* | Lugo, Spain | M |
| 18089 | *T. occidentalis* | Lugo, Spain | M |
| 18090 | *T. occidentalis* | Lugo, Spain | F |
| 21521 | *T. occidentalis* | Madrid, Spain | F |
| 21523 | *T. occidentalis* | Madrid, Spain | M |
| 21528 | *T. occidentalis* | Madrid, Spain | M |
| 21534 | *T. occidentalis* | Madrid, Spain | M |
| 21538 | *T. occidentalis* | Madrid, Spain | F |
| 21546 | *T. occidentalis* | Madrid, Spain | F |
| 21548 | *T. occidentalis* | Madrid, Spain | F |
| 21550 | *T. occidentalis* | Madrid, Spain | M |
| 21554 | *T. occidentalis* | Madrid, Spain | F |
| 21559 | *T. occidentalis* | Madrid, Spain | F |
