# Supplementary material for: Morphometric study of the bony labyrinth of the inner ear in the European moles Talpa europaea, Talpa occidentalis, and Talpa aquitania
Source: J Anat. 2025 Jul 3;248(1):71–81. doi: 10.1111/joa.70017 (PMC12682593; doi:10.1111/joa.70017)
Supplement: Supplementary file 2 — Supporting Information S2. [file JOA-248-71-s001.docx]

**SUPPORTING INFORMATION 2: 3D data preparation and protocol of fixing and sliding landmarks with AVIZO®**

The protocol we used in this work stem from the methodology of David et al. (2016) on the membranous labyrinth, which is here adapted to the bony labyrinth. The present protocol for the preparation of 3D models and has been normalized and codified so that any future comparison of bony and membranous datasets is facilitated.

1-Workfiles

Data

> SpeciesName1

> SpeciesName2

> SpeciesName3

>> Matrix

>> ScanRef

>>> LandmarksBony

>>> MaxAxesBony

>>> Other

>>> SurfacesBony

>>> VolumesBony

>>>> rv_SpeciesName3_Control_Bony ##here is the location of the STL ASCII 3D model of the bony labyrinth##

2- Preparation of the 3D model beforehand the landmark procedure

Optionnal: If necessary, the 3D model is decimated before being cut into six anatomical parts with Geomagic. The torus of each semicircular canal is composed of a slender tubular part that generally accounts at least for half the total length of the torus. The torus is also composed of a utricular part, an ampulla part and, for the vertical ducts, of a common crus part. Anterior semicircular canals (ASC) are composed of an anterior slender duct (Sa), a common crus (CC), an anterior utricle (Ua--> only available through the membranous) and an anterior ampulla (Aa). Posterior semicircular canals (PSC) are composed of a posterior slender duct (Sp), a common crus, a posterior utricle (Up --> membranous) and a posterior ampulla (Ap). Lateral semicircular canals (LSC) are composed of a lateral slender duct (Sl), an anterior utricle and a lateral ampulla (Al). ASC and LSC are connected through the anterior utricle, whereas ASC and PSC are connected through the common crus.

- Step 1: Polygons>Boundaries>Create>Boundary from spline

This step identifies and creates the anatomical boundaries, as follows:

Anterior ampulla/Slender ASC

Posterior ampulla/Slender PSC (if crus commune secundaria, stop the cut at the LSC-PSC contact)

Lateral ampulla/Slender LSC

Slender ASC/CC

Slender PSC/CC

CC/Vestibule

Slender LSC/Vestibule (if crus commune secundaria, stop the cut at the LSC-PSC contact)

Cochlea/Vestibule (start above the vestibular fenestra, run under the saccule, follow the saccule boundary until the cochlear fenestra, and reach the starting point)

- Step 2: Separation of the different anatomical parts

Duplicate our objet 5 times

For each of the six objets (ASC, PSC, LSC, CC, Vest, Cochlea): Select components (Bounded components) > Reverse > Delete

Save the three SC: in ScanRef>SurfacesBony under the names rs_SpeciesName_Sa_Bony, rs_SpeciesName_Sp_Bony and rs_SpeciesName_Sl_Bony.

Select the three CSC and click Polygons>Fill Holes>Fill All>Flat and complete, APPLY and OK. Save the three CSC: in ScanRef>VolumesBony under the names rv_SpeciesNames_Sa_Bony, rv_SpeciesNames_Sp_Bony and rv_SpeciesNames_Sl_Bony.

Select the vestibule and cochlea, click Polygons>Fill Holes>Fill All>Flat and complete, APPLY and OK. Save the vestibule and cochlea: in ScanRef>VolumesBony under the names rv_SpeciesNames_Vest_Bony and rv_SpeciesNames_Coch_Bony.

Duplicate the CC. In the first CC, the contact area with the slender SCP is filled with Polygons>Fill Holes>Fill single>Flat and complete by clicking in the area and then in Fill single. Save the object in ScanRef>SurfacesBony under the name rs_SpeciesName_Cca_Bony. Do the reverse in the othe CC and save with the name Ccp. Select Ccp, Polygons>Fill Holes>Fill all in flat and complete, APPLY, OK. Save the object in ScanRef>VolumesBony under the name rv_SpeciesName_CC_Bony.

3- Landmark protocol (with AVIZO)

Warning: always put a landmark from the ampulla to the utricle. Start by adding a landmark internally (rcs = central), then externally (res = external). For the slenders (Sa, Sp, Sl = slender of the ASC, PSC, and LSC), place the canal perpendicular to the screen plane in order to be sure to place the landmarks onto the canal.

To place a landmark: click on the landmark set, click on the icon Landmark Editor and use the tools Add, Remove, Move, or Transform.

OpenData: r_SpeciesName_Bony (in Other)

Click on the object (right window): Convert>Scan Surface To volume with the dimensions 250x250x250, APPLY

Right click on the new object, Image Processing>Skeletonization>Auto Skeleton > APPLY

Delete all the objects but except the skeleton

Open Data>VolumesBony, open all the files but Control_Bony

Creation of the landmark sets: right click on the object area, Create>Points and Lines>Landmarks

F2 to rename in rcs_SpeciesName_Sa_Bony

Duplicate (CTRL+D) and rename in rcs_SpeciesName_Sa/_Sp/_Sl/_CC/_Aa/_Sap/_Al/_Sul_Bony

and res_SpeciesName_Sa/_Sp/_Sl_Bony

- Landmark Rcs_Sa

Hide all the 3D objects but the CC and Sa. Place the first landmark at the center of the contact surface with the anterior ampulla on the Sa. Hide the Sa, make the skeleton appear and place the landmarks (>25) onto the skeleton until the surface contact with CC. Place the last landmark at the center of this surface.

- Landmark Rcs_Sp

Idem

- Landmark Rcs_Sl

Idem and place the first and last labdmarks at the vestibule sections (on Vest_Bony and not Sl_Bony)

- Landmark Rcs_CC

Display Vest and add the first landmark at the theorical crossing of the two CSC, run through the vestibule surface and place the last landmark at the center of the section (at least 5 landmarks)

- Landmark Res_Sa

Place the first landmark onto the surface of the 3D object (displaying Vest and Sa), at the center of the ampulla, on the ventral surface, below the ampulla, at the level with the crista (use the ampulla shape and the nerve arrival). Follow the crest line of the canal, until the edge of the object.

- Landmark Res_Sp

Idem

- Landmark Res_Sl

Idem but the first landmark is on the anterior surface. Follow the line crest of the canal until the conection with the vestibule.

- Landmark Rcs_Aa

Display the anterior ampulla transparent and display the skeleton. Place the first landmark onto the skeletonabove the first landmark of rcs_Sa (this position corresponds to the crista inside the ampulla). Add 2-3 landmarks in the ampulla to join the Sa.

- Landmark Rcs_SAp

Idem. Add the landmarks towards the Sp.

- Landmark Rcs_Al

Idem

- Landmark Rcs_Sul

These landmarks tie the slender LSC to the vestibule. Add a landmark in the lateral vestibul onto the skeleton where the slender LSC enters the vestibule.

- Record the landmarks: right click on the objects and register under Data>SpeciesName>ScanRef>LandmarksBony

| C_Aa | Center of the anterior ampulla |
| --- | --- |
| C_Al | Center of the lateral ampulla |
| C_Ap | Center of the posterior ampulla |
| CC | Bifurcation point of the crus commune |
| E_Aa | Pointing of the anterior crista on the external surface of the ampulla |
| E_Ap | Pointing of the posterior crista on the external surface of the ampulla |
| E_Al | Pointing of the lateral crista on the external surface of the ampulla |

 
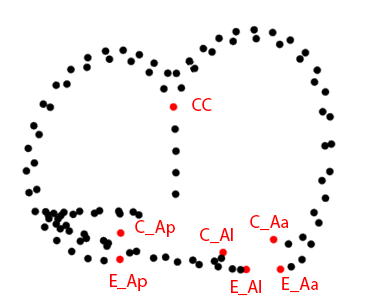


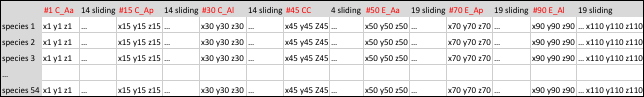


Fig. SI2. Organization of the 103 sliding landmarks and 7 fixed landmarks (in red) presented on the consensus shape.
