# Supplementary material for: Morphometric study of the bony labyrinth of the inner ear in the European moles Talpa europaea, Talpa occidentalis, and Talpa aquitania
Source: J Anat. 2025 Jul 3;248(1):71–81. doi: 10.1111/joa.70017 (PMC12682593; doi:10.1111/joa.70017)
Supplement: Supplementary file 3 — Supporting Information S3. [file JOA-248-71-s004.docx]

**SUPPORTING INFORMATION 3: R script**

library (geomorph)

fichier_landmarks <- read.csv(file.choose(),row.names=1, sep=";")

fichier_landmarks <- fichier_landmarks[,5:304]# delete qualitative data for arrays

# Convert data in array

landmarks <- arrayspecs (A = fichier_landmarks, p = 100, k=3)

# semi-landmarks

semilandmarks <- read.delim(file.choose())

# procruste analysis

procruste <- gpagen(A = landmarks, curves = semilandmarks)

# Add qualitative data

informations <- geomorph.data.frame(procruste,

espece = Test.de.répétabilité$nom.espece, numero= Test.de.répétabilité$numero.specimen, repetition = Test.de.répétabilité$X.1)

# Graphic visualisation

plotAllSpecimens(A = procruste$coords)

plot3d(x = procruste$consensus)

#PCA

ACP <- gm.prcomp(informations$coords)

plot(x = ACP, pch = 21, bg= as.factor(informations$numero))

text(ACP$x[,1]+0.002,ACP$x[,2]+0.002,labels = informations$repetition, cex=0.7)

PC <- list()

for (i in 1:11) {

PC[[i]] <- ACP$x[, i]

}

informations_jd <- data.frame(PC=PC, espece = informations$espece,

site= informations$site, sexe=informations$sexe)

colnames(informations_jd) <- c("PC1", "PC2", "PC3", "PC4", "PC5","PC6", "PC7","PC8",

"PC9", "PC10", "PC11", "Species", "Sites", "Sex")

plot.site <- ggplot(data = informations_jd, aes(x = PC1, y = PC2)) +

geom_point(size = 2, aes(shape = Species), color= "black") +

scale_colour_manual(values=palette) + scale_fill_manual(values=palette)+

labs(x = paste("PC1 :", Eigenvalues_rounded [1,]*100, "%", sep = " "),

y = paste("PC2 :", Eigenvalues_rounded [2,]*100, "%", sep = " ")) +

theme_classic()

hull.site <- informations_jd %>%

group_by(Sites) %>%

slice(chull(PC1,PC2))

plot.site <- plot.site +

geom_polygon(data = hull.site,linewidth = 0.7, aes(colour= Sites, fill= Sites ), alpha = 0.5) +

theme(plot.title = element_text(hjust = 0.5))

Eigenvalues <- as.data.frame(ACP$d/sum(ACP$d))

Eigenvalues_rounded <- round(Eigenvalues, digits = 3)

palette <- c("#FF9966","#9966CC","#330066", "#FF6633", "#CC99FF","#99FF99","#006633")

palettesp <- c("#FF9966", "#CC99FF", "#99FF99")

palettesex <- c("lightpink", "lightblue")

plot.sp <- ggplot(data = informations_jd, aes(x = PC1, y = PC2)) +

geom_point(size = 2, aes(colour = Species)) +

scale_colour_manual(values=palettesp) + scale_fill_manual(values=palettesp)+

labs(x = paste("PC1 :", Eigenvalues_rounded [1,]*100, "%", sep = " "),

y = paste("PC2 :", Eigenvalues_rounded [2,]*100, "%", sep = " ")) +

theme_classic()

hull.sp <- informations_jd %>%

group_by(Species) %>%

slice(chull(PC1,PC2))

plot.sp <- plot.sp +

geom_polygon(data = hull.sp,linewidth = 0.7, aes(colour= Species, fill= Species ), alpha = 0.5) +

theme(plot.title = element_text(hjust = 0.5))

plot.sex <- ggplot(data = informations_jd, aes(x = PC1, y = PC2)) +

geom_point(size = 2, aes(colour = Sex)) +

scale_colour_manual(values=palettesex) + scale_fill_manual(values=palettesex)+

labs(x = paste("PC1 :", Eigenvalues_rounded [1,]*100, "%", sep = " "),

y = paste("PC2 :", Eigenvalues_rounded [2,]*100, "%", sep = " ")) +

theme_classic()

hull.sex <- informations_jd %>%

group_by(Sex) %>%

slice(chull(PC1,PC2))

plot.sex <- plot.sex +

geom_polygon(data = hull.sex,linewidth = 0.7, aes(colour= Sex, fill= Sex ), alpha = 0.5) +

theme(plot.title = element_text(hjust = 0.5))

#Step 1 : import dataset

fichier_landmarks <- read.csv(file.choose(), row.names=1,

sep=";")

fichier_landmarks_si <- fichier_landmarks[,5:304]

# Step 2 : search for the closest specimen to the estimated mean

landmarks <- arrayspecs (A = fichier_landmarks_si, p = 100, k=3)

findMeanSpec(landmarks)

# here specimen 32 : 2018_2242

jacland<- fichier_landmarks_si[32,] #Extraction ind.32

# Step 3 : load files and points for modelisation

# load multivariate analyses and landmarks

# semi-landmarks

semilandmarks <- read.delim(file.choose())

# procruste analysis

procruste <- procSym (landmarks)

# add qualitative data

informations <- geomorph.data.frame(procruste,

espece = fichier_landmarks$nom_espece, numero= fichier_landmarks$numero_specimen,

sexe = fichier_landmarks$sexe, site = fichier_landmarks$localite)

#PCA

ACP <- gm.prcomp(informations$coords)

specimens_lugo <- fichier_landmarks[fichier_landmarks$localite== "Lugo, Spain",]

indices_specimens_lugo <- which(fichier_landmarks$localite == "Lugo, Spain")

coords_lugo <- procruste$coords[,,indices_specimens_lugo]

consensus_lugo <- mshape(coords_lugo)

specimens_madrid <- fichier_landmarks[fichier_landmarks$localite== "Madrid, Spain",]

indices_specimens_madrid <- which(fichier_landmarks$localite == "Madrid, Spain")

coords_madrid <- procruste$coords[,,indices_specimens_madrid]

coords_madrid<- matrix(coords_madrid, nrow = 100, ncol = 3)

consensus_madrid <- mshape(coords_madrid)

#MORPHOLOGICAL CONSENSUS

# T.occidentalis

fichier_esp <- read.csv(file.choose(), row.names=1, sep=";")

fichier_esp_si <- fichier_esp[,5:304]

landmarks_esp <- arrayspecs (A = fichier_esp_si, p = 100, k=3)

procruste_esp <- gpagen(A = landmarks_esp, curves = semilandmarks)

# T. aquitania

fichier_aqui <- read.csv(file.choose(), row.names=1, sep=";")

fichier_aqui_si <- fichier_aqui[,5:304]

landmarks_aqui <- arrayspecs (A = fichier_aqui_si, p = 100, k=3)

procruste_aqui <- gpagen(A = landmarks_aqui, curves = semilandmarks)

# T. europaea

fichier_euro <- read.csv(file.choose(), row.names=1, sep=";")

fichier_euro_si <- fichier_euro[,5:304]

landmarks_euro <- arrayspecs (A = fichier_euro_si, p = 100, k=3)

procruste_euro <- gpagen(A = landmarks_euro, curves = semilandmarks)

#Lugo

fichier_lugo <- read.csv(file.choose(), row.names=1, sep=";")

fichier_lugo_si <- fichier_lugo[,5:304]

landmarks_lugo <- arrayspecs (A = fichier_lugo_si, p = 100, k=3)

procruste_lugo <- gpagen(A = landmarks_lugo, curves = semilandmarks)

#Madrid

fichier_madrid <- read.csv(file.choose(), row.names=1, sep=";")

fichier_madrid_si <- fichier_madrid[,5:304]

landmarks_madrid <- arrayspecs (A = fichier_madrid_si, p = 100, k=3)

procruste_madrid <- gpagen(A = landmarks_madrid, curves = semilandmarks)

#Gironde

fichier_gironde <- read.csv(file.choose(), row.names=1, sep=";")

fichier_gironde_si <- fichier_gironde[,5:304]

landmarks_gironde <- arrayspecs (A = fichier_gironde_si, p = 100, k=3)

procruste_gironde <- gpagen(A = landmarks_gironde, curves = semilandmarks)

#Aveyron

fichier_aveyron <- read.csv(file.choose(), row.names=1, sep=";")

fichier_aveyron_si <- fichier_aveyron[,5:304]

landmarks_aveyron <- arrayspecs (A = fichier_aveyron_si, p = 100, k=3)

procruste_aveyron <- gpagen(A = landmarks_aveyron, curves = semilandmarks)

#PC Occidentalis

PC.esp <- procruste_esp$consensus

PC.esp <- as.matrix(PC.esp)

Mesh.esp <- warpRefMesh(jacply, jacland.matrix, PC.esp, color = "#99FF99")

#PC Aquitania

PC.aqui <- procruste_aqui$consensus

PC.aqui <- as.matrix(PC.aqui)

Mesh.aqui <- warpRefMesh(jacply, jacland.matrix, PC.aqui, color = "#FF9966")

#PC Euro

PC.euro <- procruste_euro$consensus

PC.euro <- as.matrix(PC.euro)

Mesh.euro <- warpRefMesh(jacply, jacland.matrix, PC.euro, color = "#CC99FF")

#PC Lugo

PC.lugo <- procruste_lugo$consensus

PC.lugo <- as.matrix(PC.lugo)

Mesh.lugo <- warpRefMesh(jacply, jacland.matrix, PC.lugo, color = "#99FF99")

#PC Madrid

PC.madrid <- procruste_madrid$consensus

PC.madrid <- as.matrix(PC.madrid)

Mesh.madrid <- warpRefMesh(jacply, jacland.matrix, PC.madrid, color = "#006633")

#PC Gironde

PC.gironde <- procruste_gironde$consensus

PC.gironde <- as.matrix(PC.gironde)

Mesh.gironde <- warpRefMesh(jacply, jacland.matrix, PC.gironde, color = "#FF6633")

#PC Aveyron

PC.aveyron <- procruste_aveyron$consensus

PC.aveyron <- as.matrix(PC.aveyron)

Mesh.aveyron <- warpRefMesh(jacply, jacland.matrix, PC.aveyron, color = "#FF9966")

#Linear model

procruste <- procSym (landmarks)

Y = vecx(procruste$rotated, byrow= TRUE)

dataset <-list(Y=as.matrix(fichier_landmarks))

fit <- mvols(Y~1, data = dataset)

data <- list (coords= Y,sp = as.factor(fichier_landmarksinf$nom_espece), habitat = as.factor(fichier_landmarksinf$localite),

sexe= as.factor(fichier_landmarksinf$sexe), size= procruste$size)

fitb <-mvols(coords~habitat + sp + size + sexe, data=data, method= "PL-LOOCV")

#MANOVA AND PAIRWISE

aov <-manova.gls(fitb, nperm = 999, test="Wilks", verbose=TRUE)

pairwise.glh (fitb, term= "habitat", test="Pillai", adjust="holm", nperm=500, verbose = TRUE)

#DFA

dfa <- mvgls.dfa(fitb)

plot(dfa)
