# Supplementary material for: Morphometric study of the bony labyrinth of the inner ear in the European moles Talpa europaea, Talpa occidentalis, and Talpa aquitania
Source: J Anat. 2025 Jul 3;248(1):71–81. doi: 10.1111/joa.70017 (PMC12682593; doi:10.1111/joa.70017)

**SUPPORTING INFORMATION 4**: **Results of the PCA conducted performed on the morphometric data of the bony labyrinth.** The different sexes are visualized by convex hulls and points of different colours.


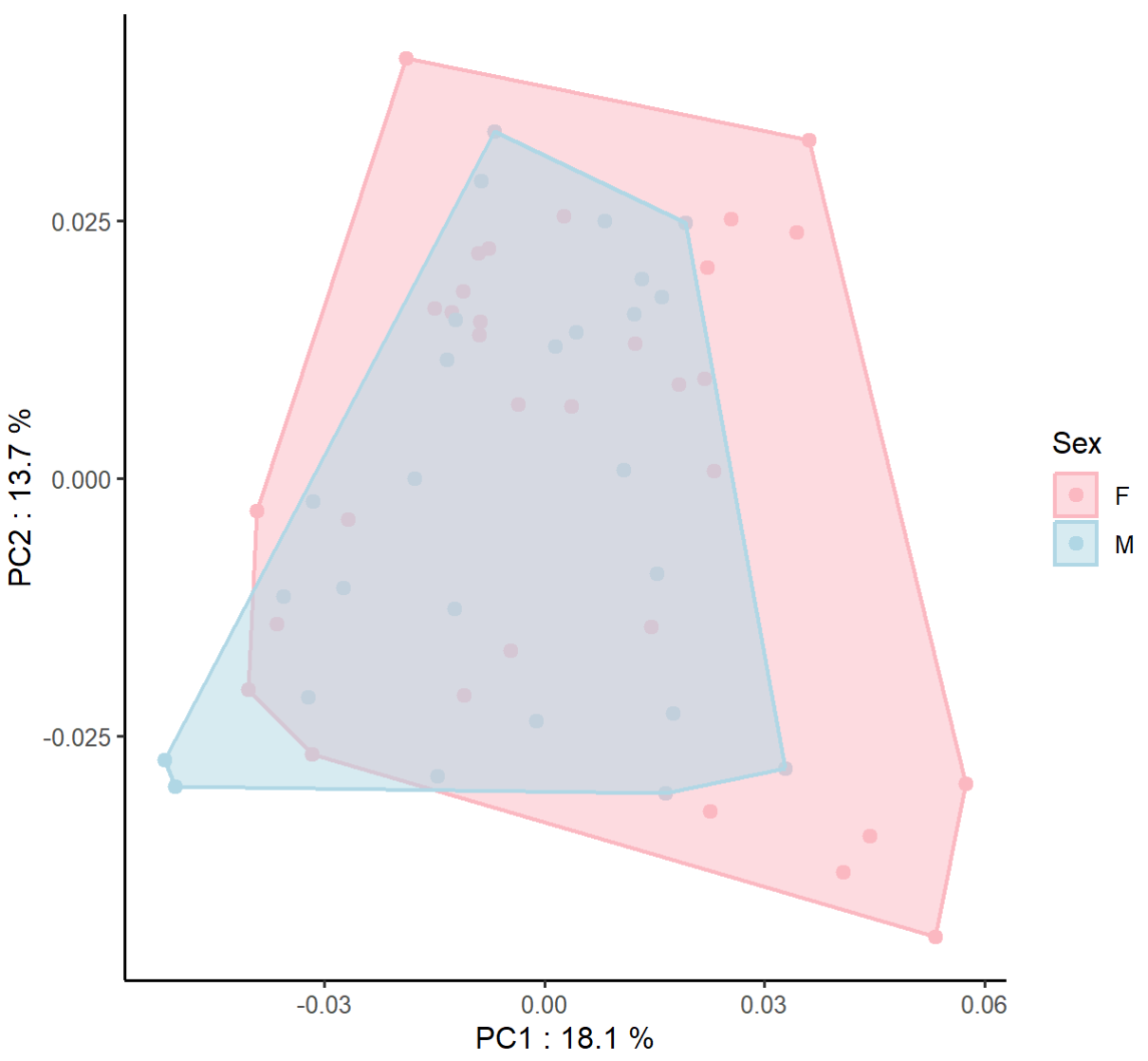

Supplement: Supplementary file 4 — Supporting Information S4. [file JOA-248-71-s003.docx]
